# Transactions of the Homœopathic Medical Society of Pennsylvania

**Published:** 1887-03

**Authors:** 


### BOOK NOTICES AND REVIEWS.

### Transactions of the Homœopathic Medical Society of Pennsylvania.

This Report of the Twenty-second Annual Session held at Philadelphia, September 20th to 23d, 1886, was issued in December. It contains the usual reports of the minutes, and then are given in the order of their occurrence many valuable papers. Among those that interested us were: A paper by Dr. Fornias, of Philadelphia, on the "Differences between Metallic Gold and its Salts," from a therapeutic standpoint; a comparison of "Belladonna, Agaricus, and Borax," by Dr. Cranch, of Erie; "Some Notes on Hay Fever," by Dr. Horace F. Ivins, giving an interesting cure with Allium cepa. Unfortunately, this paper is marred by the recommendation of old-school measures—such as Cocaine and Galvano-cautery—which the Doctor found it necessary to use.

This means, of course, that the Doctor did not find the indicated remedy and so set out to *suppress* the manifestations of the disease with these anti-homœopathic measures.

"Clinical Cases," by Dr. W. J. Martin, of Pittsburg, contains an instructive cure of a cough by Manganum met. 12th. The indication was: *the cough is always better when lying down. It will stop when the patient lies down and not trouble her until she rises.*

There are many other interesting articles in this volume, but we have not the space to devote to them. W. M. J.

